# A Narrative Review of the Coronavirus Disease 2019 Response in the Kingdom of Bahrain

**DOI:** 10.1016/j.curtheres.2024.100733

**Published:** 2024-02-01

**Authors:** Manaf AlQahtani, Jaleela Sayed Jawad, Abdulla AlAwadhi, Basma Mahmood Al Saffar, Ejlal Faisal AlAlawi, Hashim Hadi Sayed Adnan, Hammam Haridy, Jean Joury, Graciela del Carmen Morales

**Affiliations:** 1 Royal College of Surgeons in Ireland, Adliya, Kingdom of Bahrain; 2 Bahrain Defence Force Hospital, West Riffa, Kingdom of Bahrain; 3 National Taskforce for Combating the Coronavirus (COVID-19), Manama, Kingdom of Bahrain; 4 Primary Healthcare Centers, Sanabis, Kingdom of Bahrain; 5 Public Health Directorate, Ministry of Health, Manama, Kingdom of Bahrain; 6 Primary Health Centers, Supreme Health Council, Manama, Kingdom of Bahrain; 7 Medical & Scientific Affairs, Pfizer Gulf LLC, FZ Dubai, United Arab Emirates; 8 Medical & Scientific Affairs, Emerging Markets and China, Pfizer Vaccines, San Jose, Costa Rica

## Abstract

**Background:**

The Kingdom of Bahrain has reported more than 696,000 cases of coronavirus disease 2019 (COVID-19) and 1548 associated deaths as of December 26, 2022.

**Objectives:**

To better inform responses to future public health threats, this narrative review documents the challenges and responses to the COVID-19 pandemic in the Kingdom of Bahrain.

**Methods:**

A PubMed search was conducted focusing on severe acute respiratory syndrome or COVID-19 in Bahrain. Additional relevant references were also included from the authors’ personal reference collections.

**Results:**

The search indicated that Bahrain achieved well-established control of the pandemic through robust public health measures, including an early, comprehensive vaccination program. Bahrain was among the first countries to grant emergency authorization for COVID-19 vaccines; as of December 2022, nearly 73% of the eligible population has been fully vaccinated, and approximately 60% has been boosted. Low case rates in recent months highlight Bahrain's successful response to the COVID-19 pandemic.

**Conclusions:**

Early organization, robust and systematic protective measures, and a comprehensive vaccination program were key components of the Kingdom's response to the pandemic; traveler quarantines and attempts to combat misinformation were of little or no benefit. These lessons provide guidance for future preparedness to minimize the public health impacts of another pandemic. (*Curr Ther Res Clin Exp*. 2024; XX:XXX–XXX).

## Introduction

The devastating coronavirus disease 2019 (COVID-19) pandemic caused by the novel coronavirus, severe acute respiratory syndrome coronavirus 2, has led to >656 million infections and >6.6 million deaths globally as of December 26, 2022.^1^ Countries had to react to the public health threat and pressure on health systems with rapid establishment of guidelines and protocols as the pandemic evolved. It is important to document these reactions, and their relative successes and failures, to better manage future public health threats. This article aims to provide an overview of the COVID-19 challenges and responses in the Kingdom of Bahrain, using the sequence of events and countermeasures to identify the effectiveness of the latter as well as to discover areas with the potential for improvement. To accomplish this, a PubMed search for literature related to COVID-19 and the Kingdom of Bahrain was conducted to include information describing the activity of the Kingdom, including in response to Omicron variants. In addition, relevant references from the authors’ personal reference collections were included.

## The COVID-19 Pandemic in the Kingdom of Bahrain

**Fig 1:**
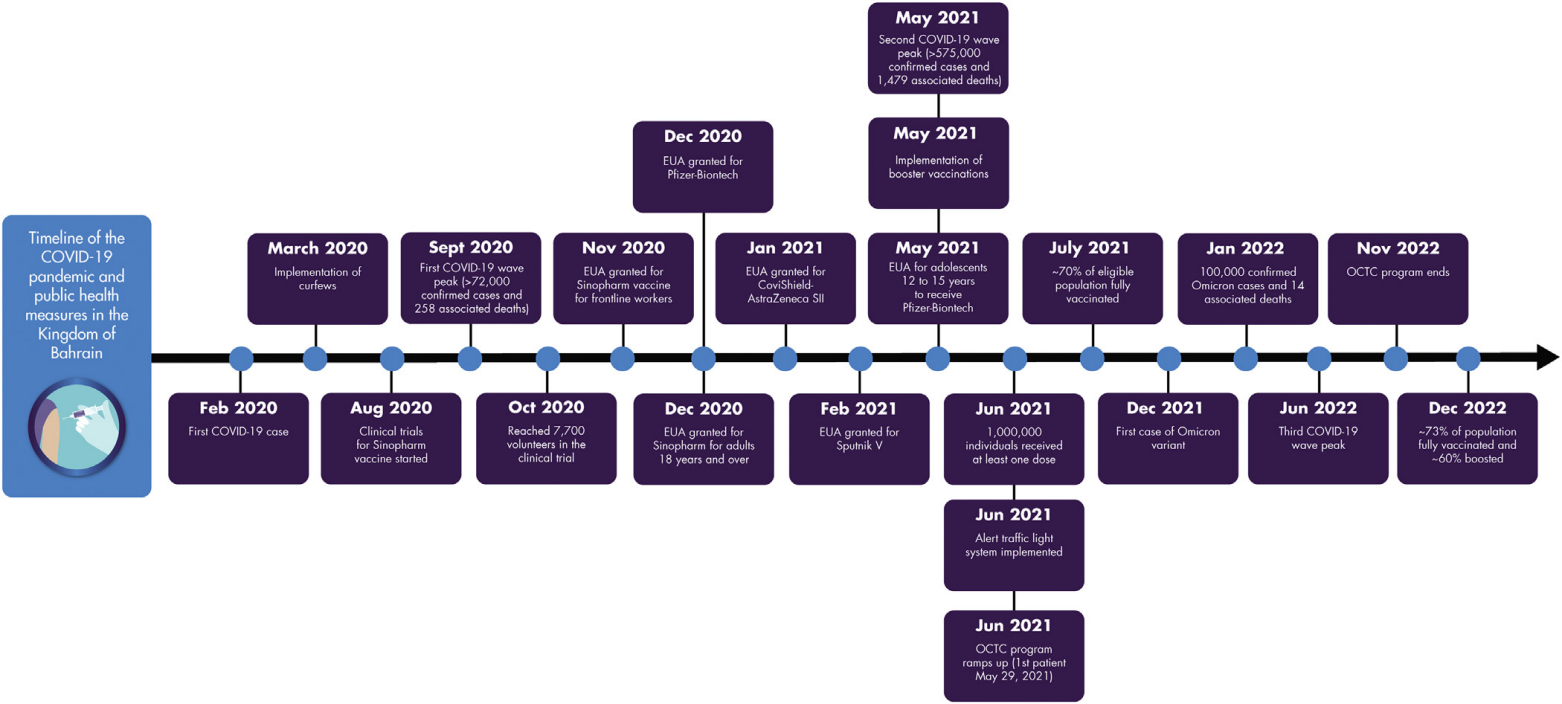
Timeline of coronavirus disease 2019 (COVID-19) pandemic and public health responses in the Kingdom of Bahrain. EUA = emergency use authorization; OCTC = Outpatient COVID-19 Therapeutics Clinic; SII = Serum Institute of India.

A timeline-based overview (Figure 1) and summary table (Table) of key events in the COVID-19 pandemic in the Kingdom of Bahrain are provided, with details on the 3 major waves of COVID-19 infections and the Kingdom's responses below.

**Table:** Summary of coronavirus 2019 (COVID-19)–related key dates in the Kingdom of Bahrain.

COVID-19–related event	Key date
First COVID-19 case	February 2020
Implementation of curfews	March 2020
Clinical trials for Sinopharm vaccine	August 2020
First COVID-19 wave peak	September 2020
Total of >72,000 confirmed COVID-19 cases and 258 associated deaths	September 2020
Emergency use authorization for COVID-19 vaccination	
Frontline healthcare workers	November 2020
General population, adults age >18 y	December 2020
General population, adolescents age 12–17 y	May 2021
Implementation of vaccine booster	May 2021
Second COVID-19 wave peak	May 2021
Total of >575,000 confirmed COVID-19 cases and 1479 associated deaths	May 2021
Outpatient COVID-19 Therapeutics Clinic initiated	May/June 2021
COVID-19 alert traffic light system	June 2021
Nearly 70% of eligible population fully vaccinated (2 doses)	July 2021
First Omicron variant case	December 2021
Total of 100,000 confirmed Omicron cases and 14 associated deaths	January 2022
Third COVID-19 wave peak	June 2022
Outpatient COVID-19 Therapeutics Clinic program shuttered	November 2022
∼73% of population fully vaccinated and ∼60% boosted	December 2022

### COVID-19 arrives in Bahrain: February 2020 to October 2020

**Fig 2:**
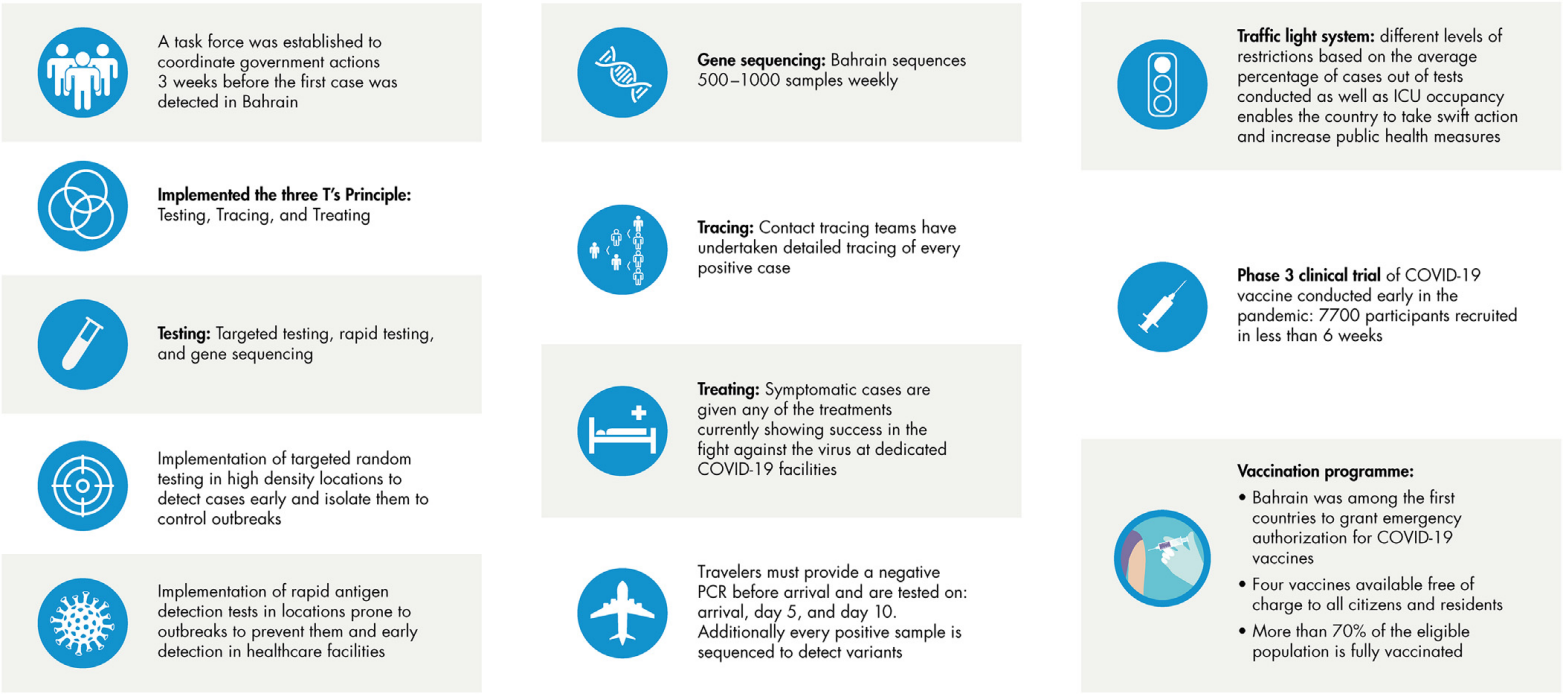
Early responses and measures against coronavirus disease 2019 (COVID-19) in the Kingdom of Bahrain. ICU = intensive care unit; PCR = polymerase chain reaction. Source: Kingdom of Bahrain Ministry of Health.^12^

Among the key features of the Kingdom's response to COVID-19 that likely contributed to its success was early preparedness. As described in Figure 2, the National Task Force for Combating COVID-19 was established several weeks before the first case of COVID-19 was identified in Bahrain. The Task Force had a broad mission relevant to all facets of the pandemic response: assess the epidemiological situation, prepare the guidelines and recommendations that should be established, monitor disease progression, prevent transmission, and treat COVID-19 cases.

The first confirmed case of COVID-19 in the Kingdom of Bahrain was identified February 24, 2020, from a school bus driver who had traveled back from Iran.^2^ Once this case was identified, public health measures recommended by the Task Force were widely implemented across the Kingdom and adjusted rapidly as needed. For example, in late March 2020, members of parliament approved a curfew to ban citizens from leaving their homes during the evening until the next morning.^3^^,^^4^ Curfews were also issued in other nations, including Saudi Arabia, United Arab Emirates (UAE), and Kuwait.^4^ During the first half of 2020, Bahrain also implemented the unique 3T program (Testing, Tracing and Treatment), with a goal of maximizing the rates of testing, contact tracing, and early treatment (Figure 2). Testing included random testing in targeted high population density locations and in locations vulnerable to outbreaks, such as schools and hospitals. Contact tracing teams worked on every positive case, and all positive cases were quarantined.^5^ Travelers into Bahrain were required to provide a negative polymerase chain reaction test before arrival and were tested on arrival, and positive samples underwent gene sequencing. The number of beds in the national health care system was doubled through the construction of field intensive care units (ICUs); a 130-bed field ICU was created in just 7 days.^5^ By the end of September 2020, the first wave of infection in Bahrain had peaked, such that 7 months after the first reported case, there had been >72,000 confirmed cases of COVID-19 and 258 related deaths.^1^

### Second wave of COVID-19 in Bahrain: October 2020 to May 2021

Although the first wave of COVID-19 infections had peaked, the infection rate of COVID-19 remained dangerously high. During the first wave, vaccines had been developed and rapidly brought to clinical trials (Figure 1). The first clinical trials, for the BBIBP-CorV (Sinopharm) vaccine, began in August 2020 (Figure 1 and Table), and by October 2020 there had been a Phase 3 trial with 7700 local participants.^6^ During November 2020, emergency use authorization (EUA) was granted for the Sinopharm vaccine. Rapid adoption of the vaccines under EUA was in line with the Kingdom's intent of early preparedness, and indeed, Bahrain was among the first countries to grant the COVID-19 vaccines EUA status.^7^ Vaccination in the general population started on December 16, 2020, shortly after EUA approval of the BNT162b2 mRNA and BBIBP-CorV vaccines on December 3, 2020, and December 12, 2020, respectively.^8^ The timeline of Bahrain's vaccination program was similar to or earlier than other countries in the region; vaccination among the general population in Saudi Arabia, Qatar, and UAE commenced in December 2020 following EUA.^7^^,^^9^^,^^10^ Shortly after, during February 2021, Iran started administering COVID-19 vaccines among frontline health care workers followed by the general population.^11^

Four vaccines against COVID-19 are currently available free of charge for adults in Bahrain: BBIBP-CorV (Sinopharm; Beijing, China), BNT162b2 (Cominarty; Pfizer; New York, New York, and BioNTech; Mainz, Germany), ChAdOx1-SARS-COV-2/AZD1222 (CoviShield; AstraZeneca/Serum Institute of India, Pune, India), and Gam-COVID-Vac (Sputnik V; Gamaleya Research Institute of Epidemiology and Microbiology; Moscow, Russia). BNT162b2 is available for those aged 5 years and older.^12^

The Bahrain vaccination program was enabled by expansion of the already present and robust routine vaccination platform infrastructure; Bahrain has had >95% coverage for all childhood vaccines in the past 20 years.^13^ Vaccinations take place at 31 vaccination sites, which are distributed throughout the country and are geographically accessible. Mobile vaccination clinics and services are provided for the elderly and those with special needs who cannot access these vaccination sites. Additional personnel (ie, medical students and nurses) were mobilized and trained to administer vaccines and monitor adverse events (AEs). There was also proactive identification and preparation of cold chain requirements to store and disseminate vaccines; Bahrain improved upon the already successful cold chain and vaccine management practices to procure the equipment and supplies needed to properly store and administer COVID-19 vaccines.^13^

A self-reporting vaccine AE system was put in place that categorized the severity and referred the patients for secondary assessment where they could receive medical attention if required. AEs could be reported through health care providers at any health facility and through self-reporting. Vaccination is available to all citizens and residents in Bahrain, with priority given to those at increased risk of exposure according to the World Health Organization priority road map: individuals age 50 years or older, health care and frontline workers, and individuals with underlying health conditions. Ease of access to information and education regarding COVID-19 and vaccination was ensured, with a multilingual media campaign launched early in the epidemic campaign and a mobile application.

Despite the deployment of vaccines, a second, larger COVID-19 peak led to weekly case numbers as high as 20,800 during the week of May 24, 2021, whereas weekly deaths peaked at 152 at the end of May.^1^ In response, between May 27, 2021, and June 10, 2021, the Bahrain Task Force announced strict new measures to prevent the spread of COVID-19.

In response to this new wave of cases, 2 new programs were instituted. First, the Kingdom initiated the Outpatient COVID-19 Therapeutics Clinic (OCTC) program, which saw its first patient on May 29, 2021. The OCTC program offered several monoclonal antibody therapies and nirmatrelvir/ritonavir (Paxlovid; Pfizer Inc) to patients depending on the results of an immediate evaluation. One component was a call center and hotline dedicated to trying to gather as much information as possible on every COVID-19–positive patient in Bahrain and to providing information, scheduling appointments, and collecting follow-up information such as treatment efficacy, side effects, and AEs. The other key component was a combination of mobile and fixed clinics to diagnose and treat patients. Briefly, potential patients would be registered, polymerase chain reaction tested, and offered treatment within 24 hours if a positive test occurred. Patient demographic/risk factor data and medication use would be collected, and after assessment by a doctor, the appropriate treatment option would be offered. In the case of nirmatrelvir/ritonavir, medication interactions would be assessed. The clinics included a dedicated pharmacy to ensure the correct medication was provided. A computerized system was established to speed up the process and maximize safety and efficiency without sacrificing patient-doctor interactions. Once implemented, OCTC treated an average of approximately 100 patients per day, reducing the COVID-19–related burden on the rest of the health care system. The OCTC program ran until after the third wave of COVID-19 had subsided (see below).

On June 30, 2021, the Task Force announced the second program, a COVID-19 alert “traffic light” system, with different levels of restriction based on the average percentage of cases out of tests conducted, as well as ICU occupancy.^12^ The traffic light system enabled the country to take action swiftly and increase public health measures should case rates begin increasing again.

As early as July 2021, more than 1 million people in Bahrain had received 2 doses of vaccine (ie, nearly 70% of the eligible population was fully vaccinated). By contrast, as of August 16, 2021, the first and second doses of vaccine were administered in 13.1% and 5.2% of the total Iranian population.^14^ Notably, the daily death rate, when including those deaths not officially reported but likely to be COVID-19–related, was ∼0.03 deaths/100,000 people in Bahrain on August 16, 2021, whereas it was ∼1.23/100,000 in Iran.^15^

### Advent of Omicron variants in Bahrain: June 2021 to May 2022

At the first peak of infections in May 2021, there were more than 20,000 cases per week. From the end of June 2021 to December 2021, Bahrain experienced low case numbers of COVID-19; however, there has been a recent increase in infections with a peak of >51,000 cases reported during the final week of January 2022.^1^ It is likely this increase was related to the advent of Omicron variants.

While continuing surveillance to monitor severe acute respiratory syndrome coronavirus 2 variants of concern, the Bahrain Ministry of Health announced on December 11, 2021, that a case of the Omicron variant was detected in an individual arriving from abroad.^12^ On February 3, 2022, the ministry reported that, since January 2022, there had been 100,000 Omicron cases and 14 deaths. In comparison, the Delta variant caused 112,000 cases and 787 deaths in 2021. The low case fatality rate observed with Omicron (0.01%) highlights the effectiveness of the vaccination campaign in Bahrain in reducing deaths, although the low case fatality rate can be partially attributed to the fact that infection with the Omicron variant was less deadly in general.

**Fig 3:**
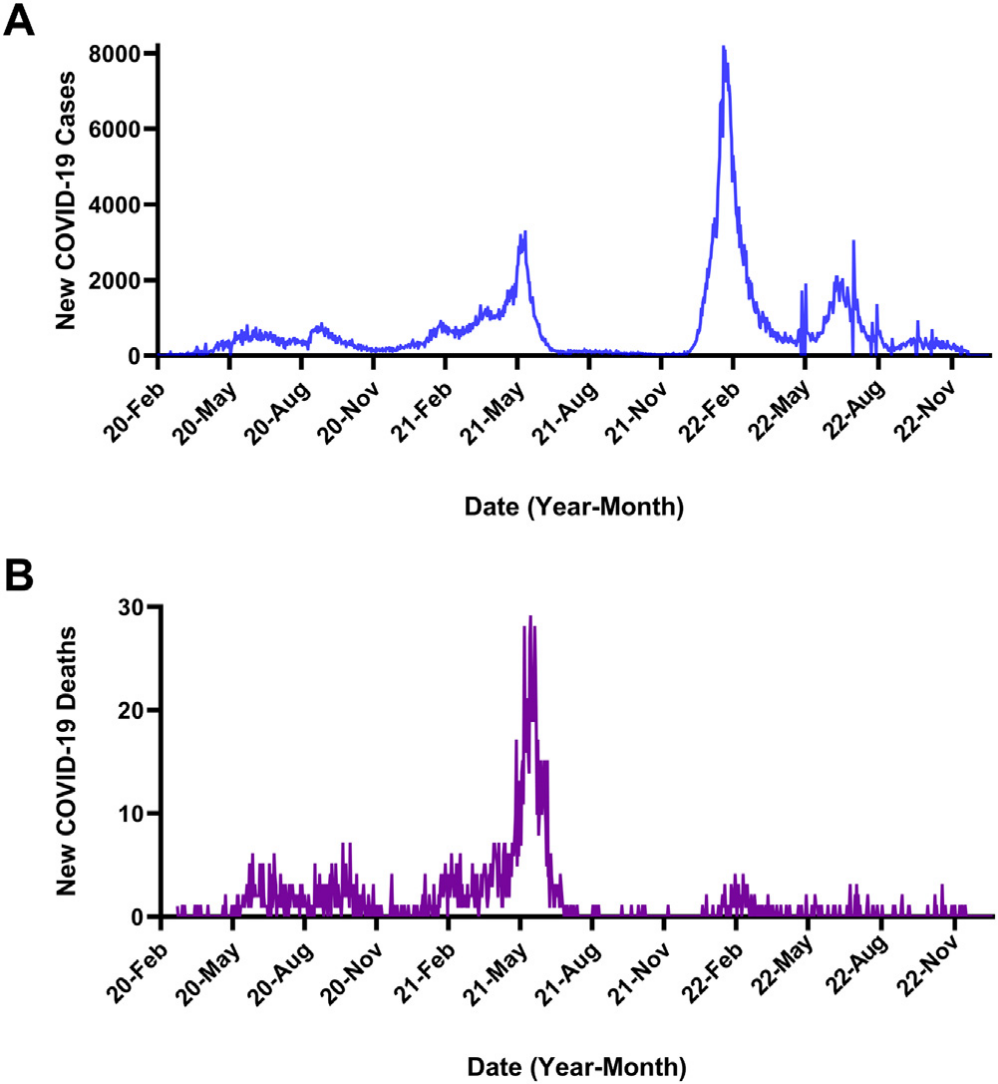
Incidence of coronavirus disease 2019 (COVID-19) in the Kingdom of Bahrain. (A) New cases. (B) New deaths. Data are available at https://covid19.who.int/WHO-COVID-19-global-data.csv.

Regardless of the root cause(s), the number of associated deaths has remained low (Figure 3). As of May 13, 2022, Bahrain had >575,000 confirmed COVID-19 cases and 1479 associated deaths.^1^ By contrast, Bahrain's total number of confirmed COVID-19 cases and deaths were fewer than those reported in other countries in the region.^16^ Although reported cases and deaths were fewer in Qatar than in the Kingdom, as of May 13, 2022, Saudi Arabia had 758,361 cases and 9111 deaths, UAE had 901,809 cases and 2302 deaths. Iran, which lagged the other nations slightly in introducing its vaccination program, had >7,200,000 cases and >140,000 deaths.

### Third COVID-19 wave in Bahrain: June 2022 to December 2022

The third wave of COVID-19 peaked during June 2022 at almost 13,000 new cases in a week, about 24% of the peak case rate of the second wave. Like the second COVID-19 wave, and unlike the first wave, the third wave was not accompanied by a large spike in the weekly death rate. From July 2022 through the end of the calendar year 2022, both new cases and new deaths remained relatively low (Figure 3); under these conditions, the OCTC program was discontinued at the end of November 2022.

## Areas of Success in the Kingdom of Bahrain's COVID-19 Response

The successful (or robust) public health response to the COVID-19 pandemic in Bahrain has been documented in different publications. An appraisal of the COVID-19 response in the Gulf according to World Health Organization criteria reported that Bahrain was fully compliant with all 11 pillars of the modified strategic response measures.^17^ These measures included country-level coordination planning and monitoring; risk communication and community engagement; surveillance, rapid response, and case investigation; development of strategies around border points of entry, international travel, and transport; infection prevention and control strategies; active management of COVID-19 cases; sufficient operational support and logistics; management of essential health services and systems; application of public health measures; conducting research during the pandemic; and care for migrants and expatriate workers.^17^ A study of 142 emergency physicians from 3 major hospitals in Bahrain found that, in general, physicians showed good knowledge, positive attitude, and reasonable practice skills to combat COVID-19.^18^ It should be noted that high rates of asymptomatic infections have been reported in Bahrain; 1 study of 320 index cases and 1289 positive contacts reported that 50% of index cases and 87% of positive contacts were asymptomatic.^19^ Another study reported that 116 of 188 positive patients (85%) were asymptomatic.^20^ These observations highlight the importance of vaccination, testing, and contact tracing as part of a comprehensive antipandemic program.

Our information indicates the vaccine program has been a successful component of the Kingdom's response plan. Implementation of vaccine rollout has resulted in vaccination of more than 73% of the population with 2 doses of a COVID-19 vaccine. A COVID-19 booster vaccine program was implemented, initially in individuals older than age 50 years, frontline workers including health care workers, and those with underlying health conditions, and then to the general population, for all currently available vaccines. Nearly 60% of the eligible population has received a booster dose. Bahrain has among the lowest case fatality rates in the entire Eastern Mediterranean Region.^21^ High vaccination rates have indirect benefits of reducing the likelihood of needing to quarantine or isolate in the future. This may be a critical, if sometimes overlooked, aspect of the vaccine program, given the limited efficacy of the traveler quarantine program.^22^

## Areas Needing Improvement in the Kingdom of Bahrain's COVID-19 Response

Improvements in treatment of severe/symptomatic cases are necessary. Although not unique to Bahrain, treatment for COVID-19 has mostly been limited to palliative care. Symptomatic cases were treated with the best available treatments at the time, and research was conducted in-country, such as a pilot clinical trial with convalescent plasma. Analyses of data from the OCTC program suggest nirmatrelvir/ritonavir may represent a step toward that improvement, although more granular information is required to confirm this.

Overall, and not entirely specific to the Kingdom of Bahrain, efforts should be made to improve the collection and distribution of accurate information. One critical aspect of this is the collection and reporting of all-cause and specific-cause mortality rates. Excess mortality is defined as the difference between the expected number of deaths based on pre-pandemic mortality rates and the actual number of all-cause deaths reported during the pandemic; this can potentially demonstrate the full influence of COVID-19 and nation-specific responses to it, especially when official reported data are lacking.^23^ For example, 1 report attempting to estimate pandemic-related excess mortality at the global and national levels noted that by the end of the year 2021, there were 5.94 million reported COVID-19 deaths and an estimated 18.2 million excess deaths (3.07 per reported death).^23^ However, a lack of specific-cause mortality data for some time periods and some countries, as well as differences in reporting and definitions of cause of death between countries, prevented the authors from producing a numerical estimate of deaths due directly to COVID-19. The authors did conclude that a large fraction of the excess deaths are likely related to the COVID-19 pandemic.^23^ Their results for the Kingdom of Bahrain for the same time period estimated an excess mortality rate of about 2.81, in line with both the global rate and those of many nearby countries but also indicating that Bahrain too may not have the most accurate picture of the effects of the pandemic and its responses to it.^23^ Some information can be gained from these data. Comparing the most recent excess mortality data available between Bahrain and Iran, which was generally slower to respond and had much less vaccine coverage over the period covered in this Review, suggests that Bahrain's responses to the pandemic were more successful than those of Iran. The growth in total number of deaths (including excess mortality) by summer 2021 had flattened in Bahrain, whereas in Iran it was approximately 1 more year before such success was achieved.^15^ Distinguishing the degree to which specific components were responsible for this success would require much more granular and complete data.

Not all of the public safety measures implemented per the Task Force's recommendations were deemed beneficial. Notably, a retrospective study conducted to analyze COVID-19 incidence in travelers arriving in Bahrain^22^ found that quarantining travelers was not likely to have been beneficial. It did identify the use of testing upon arrival in conjunction with other public health measures as beneficial in lowering the risk of transmission. In addition, improvements are necessary in the dissemination of accurate information. For example, 1 study assessed knowledge and attitudes of the pandemic in a potential high-infection-risk group (university students) in Bahrain.^24^ Students demonstrated a good understanding of the actions they needed to take to prevent transmission of COVID-19, including masking and avoiding contact, but only a small majority (58.4%) were aware vaccines were available. In addition, 78.0% used social media for their main source of knowledge, and not coincidentally, more students who were willing to answer questions on the commonly cited COVID-19 misconceptions (eg, COVID was manmade, can be contained with herd immunity, or can be prevented with common vitamin supplements) believed those misconceptions.^24^ Because these beliefs could foster distrust in an official pandemic response, and thus interfere with vaccine uptake or treatment distribution in a future pandemic, it is recommended that investment in efforts to improve outreach and combat disinformation campaigns should be prioritized as part of an overall response plan.

This study had several inherent strengths and limitations. Its format as a narrative review provides a natural framework for examining the temporal relationships between the pandemic and the Kingdom's responses to it, potentially revealing which responses were beneficial. The study was limited by the lack of availability of some potentially useful data, which reduced our ability to identify which specific components were most/least successful. Also, the utility of the responses by the Kingdom of Bahrain to the challenges of the pandemic may not reflect their utility in other nations depending on the unique cultural and social aspects of that country and the characteristics of the infectious agent itself.

## Conclusions

Although reported cases and deaths were fewer in Qatar, Bahrain's total number of confirmed COVID-19 cases and deaths were fewer than those reported in most other countries in the region. These data suggest that, on the whole, the Kingdom of Bahrain's measures put in place to fight the pandemic were helpful, at least in limiting the death toll and preventing the Kingdom's medical services from being overwhelmed during peak infection periods. Bahrain's robust and early public health response to the COVID-19 pandemic included testing, contact tracing, and treating all cases; quarantine and other public health interventions; and a comprehensive and swift vaccination program. Following World Health Organization guidelines to explain how or why implementation of an intervention leads to effects on health behavior or status and documenting successful experiences from countries during this pandemic could contribute to adoption of best practices and future guidelines for preparedness to minimize the influences of the next public health threat.

## Declaration of competing interest

This work was sponsored by Pfizer Gulf FZ, LLC. H. Haridy, J. Joury, and G. D. C. Morales are employees of Pfizer and may hold stock or stock options. The authors have indicated that they have no other conflicts of interest regarding the content of this article.
